# Identification of Cardiac Glycosides as Novel Inhibitors of eIF4A1-Mediated Translation in Triple-Negative Breast Cancer Cells

**DOI:** 10.3390/cancers12082169

**Published:** 2020-08-04

**Authors:** Cory M. Howard, Matthew Estrada, David Terrero, Amit K. Tiwari, Dayanidhi Raman

**Affiliations:** 1 Department of Cancer Biology, University of Toledo Health Science Campus, Toledo, OH 43614, USA; cory.howard@rockets.utoledo.edu (C.M.H.); estrada.78@buckeyemail.osu.edu (M.E.); 2 Department of Pharmacology & Experimental Therapeutics, College of Pharmacy & Pharmaceutical Sciences, University of Toledo Main Campus, Toledo, OH 43606, USA; davidalejandro.terrerorodriguez@rockets.utoledo.edu (D.T.); amit.tiwari@utoledo.edu (A.K.T.)

**Keywords:** eIF4A1, c-MYC, digoxin, bufalin, rocaglamide A, TNBC, repurposing, repositioning

## Abstract

The eukaryotic translation initiation factor 4F complex (eIF4F) is a potential chemotherapeutic target in triple-negative breast cancer (TNBC). This complex regulates cap-dependent translational initiation and consists of three core proteins: eIF4E, eIF4G, and eIF4A1. In this study, we focus on repositioning compounds as novel inhibitors of eIF4A1-mediated translation. In order to accomplish this goal, a modified synthetic reporter assay was established. More specifically, a (CGG)_4_ motif, which confers eIF4A dependency, was incorporated into the 5’-leader region of a luciferase-tdTomato lentiviral reporter construct. The Prestwick Chemical Library was then screened in multiple TNBC cell lines by measuring the tdTomato fluorescent intensity. We identified several cardiac glycosides as potential inhibitors of eIF4A1-mediated translation. Based on our studies, we find that cardiac glycosides inhibit the expression of eIF4A1. To identify a potential mechanism by which this was occurring, we utilized the Integrative Library of Integrated Network-Based Cellular Signatures (iLINCS). Our pursuits led us to the discovery that cardiac glycosides also decrease levels of c-MYC. Quantitative PCR confirmed that decreases in c-MYC and eIF4A were occurring at the transcriptional level. As such, disruption of the eIF4A1-c-MYC axis may be a viable approach in the treatment of TNBC. The novel combination of rocaglamide A and digoxin exhibited synergistic anti-cancer activity against TNBC cells in vitro. The findings in this study and others are important for formulating potential combination chemotherapies against eIF4A1 in vivo. Thus, drug repositioning may be one classical approach to successfully target eIF4A1 in TNBC patients.

## 1. Introduction

Triple-negative breast cancer (TNBC) is a subtype of breast cancer that lacks detectable expression of estrogen receptor (ER), human epidermal growth factor receptor 2 (HER2), and progesterone receptor (PR). TNBCs have an increased likelihood of recurrence and mortality in the first 3 years of treatment [1,2]. This is most likely due to the propensity of TNBC to metastasize and lack of effective targeted therapies [3]. Standard neoadjuvant chemotherapy using taxane and anthracycline-based agents is currently the standard-of-care [4,5]. Several promising therapies have emerged in the field including therapeutic antibodies targeting programmed cell death protein 1 (PD-1), and its ligand PD-L1, along with poly ADP ribose polymerase (PARP) inhibitors, but these therapies unfortunately only benefit a fraction of patients [6,7]. One of the emerging targets in TNBC is the eukaryotic initiation factor (eIF) 4F complex.

The eukaryotic initiation factor (eIF) 4F complex regulates the rate-limiting step of translational initiation. This complex consists of the cap binding protein eIF4E, the large scaffolding protein eIF4G, and the DEAD-box RNA helicase eIF4A1. eIF4F assists in the recruitment of the 43S preinitiation complex (PIC) to the messenger RNA [8]. The necessity for the eIF4F complex is largely dependent on the structural diversity of the 5′-leader region [9]. Sequences that contain a high degree of secondary structures (or stem-loops) require the ATP-dependent helicase activity of eIF4A1 to unwind any folding which then allows for the successful recruitment of the PIC to the mRNA.

There has been considerable interest in the eIF4F complex and its vital connection to cancer biology. Several signaling pathways are frequently dysregulated in cancer and these feed into the mTOR complex which ultimately promotes the assembly of eIF4F through a series of phosphorylation reactions [10]. Moreover, eIF4F cancer dependency is largely due to several oncogenic mRNAs containing complex secondary structures in their 5′-leader region. Examples of eIF4F-dependent mRNAs include baculoviral IAP repeat containing 5 (BIRC5), Rho associated coiled-coil containing protein kinase 1 (ROCK1), cyclin D3 (CCND3), c-MYC, and B-cell lymphoma-2 (BCL-2) [11].

The role of eIF4F complex in primary breast tumor progression and metastasis has been studied. Knockdown of eIF4E reduced the migration and invasion of TNBC cells in vitro. Moreover, the loss of eIF4E reduced primary tumor growth and the onset of pulmonary metastasis [12]. Importantly, eIF4A1 and eIF4E were found to be independent predictors of poor outcome in ER-negative breast cancer [13]. Recently, we indicated a role for eIF4A1 in facilitating paclitaxel resistance in TNBC cell lines [14].

As the eIF4F complex immensely supports the cancer proteome to enable primary tumor progression and metastasis, there have been several pursuits to identify inhibitors that disrupt its activity. Some examples include 4E-GI-1 which prevents the protein-protein interaction between eIF4E and eIF4G [15]. In addition, there are several small molecule inhibitors (SMIs) which disrupt the activity of the eIF4A1. This includes pateamine A and hippuristanol [16,17]. Among the SMIs of eIF4A1 to date, the best characterized set of inhibitors is the rocaglates. Rocaglates clamp eIF4A1 onto RNA polypurine sequences in an ATP-independent manner. This prevents the participation of eIF4A1 in the eIF4F complex which disrupts 43S PIC recruitment and the translation of the oncogenic mRNAs [18,19]. These anti-eIF4A1 inhibitors demonstrated remarkable efficacy in the treatment of TNBC. For example, silvestrol significantly inhibited the growth of MDA-MB-231 orthotopic xenograft tumors [20]. Inhibition of eIF4A is also a promising strategy for other cancer types including pancreatic cancer [21]. New amidino-rocaglate derivatives have recently been developed with an IC_50_ value as low as 0.97 nM in MDA-MB-231 cells [22,23]. One rocaglate derivative, eFT226 (Zotatifin) has just entered a phase 1 clinical trial in patients with advanced solid tumors (NCT04092673) [24]. Despite the clear and potent anti-cancer activity of eIF4F inhibitors, none are FDA-approved currently. 

Drug repositioning or repurposing is the process by which novel uses are found for pre-existing drugs. This approach has several distinct advantages over the conventional drug development pipeline. For example, the biosafety, pharmacokinetic, and pharmacodynamic profiles of any FDA-approved drug are already known which is beneficial in the clinical trial process. One successful example of repositioning compounds as an anti-cancer agent is the use of thalidomide in the treatment of multiple myeloma [25]. Several studies have suggested that commonly used medications such as β-blockers, angiotensin-converting enzyme (ACE) inhibitors, or statins may actually reduce breast cancer mortality [26].

In this study, we modified an eIF4A1-reporter system to screen the Prestwick Chemical Library consisting of mostly FDA-approved drugs. To our knowledge, this is the first attempt to repurpose compounds as potential inhibitors of eIF4A-mediated translation. Such compounds could potentially show potent anti-cancer activity while making a quick clinical impact. In our drug screening, we identified that cardiac glycosides have the ability to inhibit eIF4A1 through a c-MYC-dependent mechanism.

## 2. Results

### 2.1. Establishment of the (CGG)_4_ Luc2-TdTomato Reporter System

**Figure 1:**
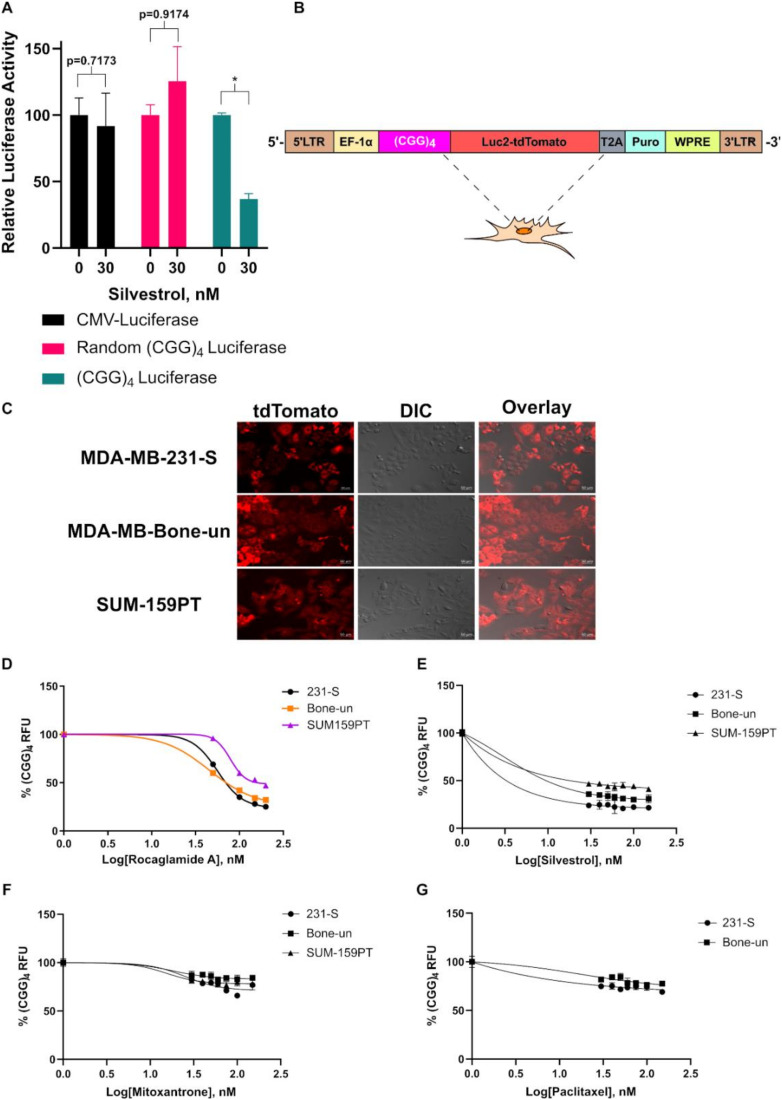
Characterization of the (CGG)_4_ Luc2-tdTomato Reporter System. (**A**). (CGG)_4_ luciferase assay in 293 cells following treatment with 30 nM silvestrol (*n* = 3). Relative luciferase activity indicates the luminescent ratio of luciferase/renilla with untreated cells set to 100% (* indicates *p* < 0.05 as analyzed by a student’s t-test). (**B**). Cartoon representation of the (CGG)_4_ Luc2-tdTomato reporter plasmid. (**C**). Live-cell fluorescent images of MDA-MB-231-S, MDA-MB-Bone-un, and SUM-159PT cells after introduction of the (CGG)_4_ Luc2-tdTomato fusion protein (*n* = 1). (**D**). (CGG)_4_ Luc2-tdTomato readings in MDA-MD-231-S, MDA-MB-Bone-un, and SUM-159PT cells following treatment with 0–200 nM rocaglamide A (*n* = 3). (**E**). (CGG)_4_ Luc2-tdTomato readings in MDA-MD-231-S, MDA-MB-Bone-un, and SUM-159PT cells following treatment with 0–150 nM silvestrol (*n* = 3). (**F**). (CGG)_4_ Luc2-tdTomato readings in MDA-MD-231-S, MDA-MB-Bone-un, and SUM-159PT cells following treatment with 0–150 nM mitoxantrone (*n* = 3). (**G**). (CGG)_4_ Luc2-tdTomato readings in MDA-MD-231-S and MDA-MB-Bone-un cells following treatment with 0–150 nM paclitaxel (*n* = 3).

The (CGG)_4_ motif located in the 5′-leader region of mRNAs has been recognized to confer eIF4A1 dependency [27]. Early studies characterizing the structural characteristics of the (CGG)_4_ motif have hypothesized the formation of a G-quadruplex. However, the actual existence of G-quadruplexes within the cell remains controversial. Recent evidence has suggested that this motif rather confers eIF4A dependency through the formation of classical secondary structures [28,29]. Irrespective of the precise nature of the secondary structure, we first confirmed the dependency of the (CGG)_4_ motif on the activity of eIF4A by employing a previously established luciferase assay [27]. Human embryonic kidney-293 (HEK-293) cells were transiently transfected with CMV-luciferase, CMV-(CGG)_4_ luciferase, or CMV-random (CGG)_4_ luciferase (matched for G/C content) and incubated with silvestrol. There was a significant reduction in the relative luciferase activity for the construct harboring the (CGG)_4_ motif in the 5′-leader region (Figure 1A). Based on these findings, we created an eIF4A1 reporter gene which harbored a (CGG)_4_ motif immediately upstream of the open reading frame of the luciferase-tdTomato fusion protein (Figure 1B). In order to track the eIF4A1 activity, we can either employ luciferase or tdTomato as the reporter depending on the contextual requirement. Following stable selection with puromycin in multiple TNBC cell lines, successful incorporation and expression of the fusion protein was confirmed by live-cell fluorescent microscopy (Figure 1C). To confirm dependency of the reporter protein on the activity of eIF4A1, the concentration-dependent response of two known eIF4A inhibitors, rocaglamide A and silvestrol, was tested. Rocaglamide A and silvestrol potently reduced Luc2-tdTomato expression levels in a concentration-dependent manner (Figure 1D,E). To further confirm the specificity of our system to eIF4A1, we tested the inhibitory response following the incubation with two cytostatic drugs that are not known to inhibit the helicase activity of eIF4A1, mitoxantrone, and paclitaxel. Both compounds reduced Luc2-tdTomato expression by only 20% in the same concentration range (Figure 1F,G). In all, our results suggest that the establishment of a reporter system which does indeed confer eIF4A dependency.

### 2.2. Prestwick Chemical Library Screen

**Figure 2:**
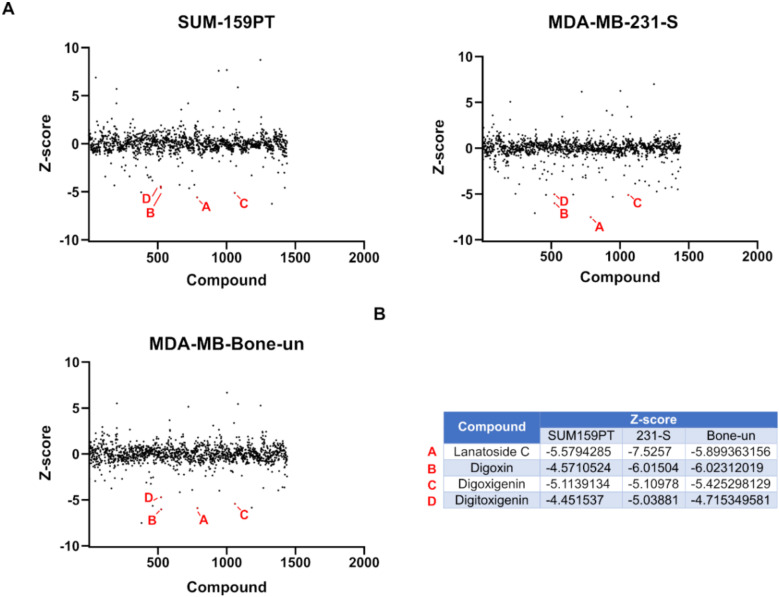
Prestwick Chemical Library Screen. (**A**). Z-scores for each compound from the Prestwick Chemical Library were calculated for their ability to reduce expression levels of (CGG)_4_ Luc2-tdTomato in MDA-MB-231-S, MDA-MB-Bone-un, and SUM-159PT cells (*n* = 1). (**B**). Individual Z-scores of lanatoside C, digoxin, digoxigenin, and digitoxigenin from each cell line.

We screened the Prestwick chemical library in order to reposition compounds that potentially inhibit eIF4A1-mediated mRNA translation in TNBC cells. This library contains mostly FDA-approved drugs with known bioavailability and toxicity profiles. To further confirm accuracy of each hit, we decided to screen the library in differing TNBC reporter cell lines. Z-scores, or the number of standard deviations away from the mean fluorescence, was determined for each compound (Figure 2A). When comparing the results between cell lines, cardiac glycosides significantly inhibited Luc2-tdTomato expression in our eIF4A1 reporter system. The individual Z-scores of each compound are listed (Figure 2B). Cardiac glycosides are a class of compounds which are known to inhibit the Na^+^/K^+^-ATPase. These compounds have been used clinically for decades to treat heart failure and atrial arrhythmia [30]. Based on these findings, we further pursued the cardiac glycoside digoxin and its derivative bufalin in our studies. The anti-cancer activity of digoxin has been identified previously [31]. Moreover, bufalin has been shown to inhibit the growth of tumors in a xenograft model of breast cancer [32]. Bufalin has also been reported to inhibit the PI3K-Akt-mTOR pathway which is known to contribute to eIF4F assembly [33].

### 2.3. Cardiac Glycosides Inhibit eIF4A-Mediated Translation in Triple-Negative Breast Cancer Cells

**Figure 3:**
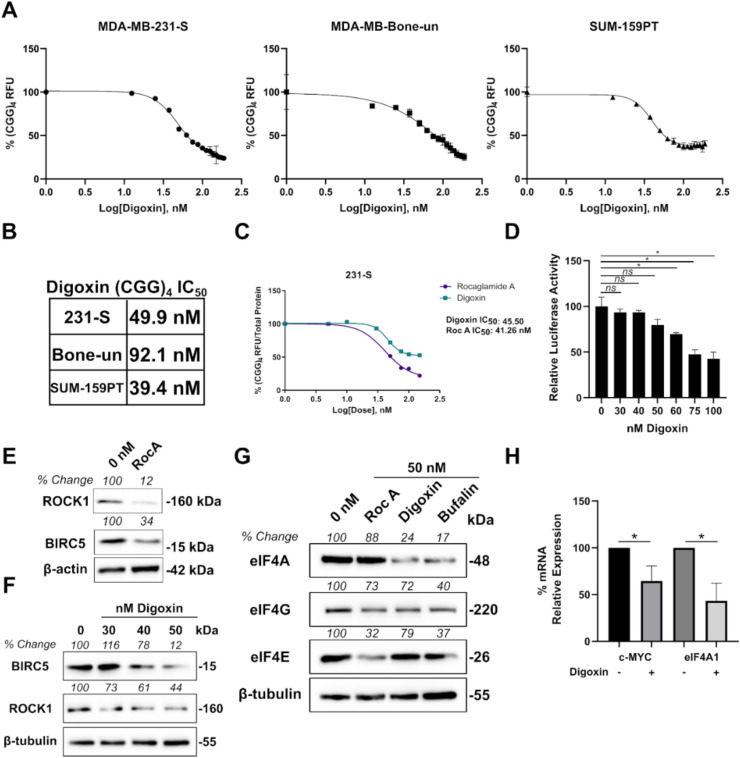
Cardiac glycosides inhibit eukaryotic translation initiation factor 4A complex (eIF4A)-dependent translation in triple-negative breast cancer (TNBC) cells. (**A**). (CGG)_4_ Luc2-tdTomato readings in MDA-MD-231-S, MDA-MB-Bone-un, and SUM-159PT cells following treatment with 0-187.5 nM digoxin (12.5 nM increments). (**B**). Calculated (CGG)_4_ IC_50_ values for each cell line. (**C**). (CGG)_4_ Luc2-tdTomato/total protein readings (A595) in MDA-MD-231-S cells following treatment with either 0-150 nM digoxin or rocaglamide A. (**D**). CMV-Luciferase/RTK Renilla expression readings in 293 cells following treatment with 0–100 nM digoxin (* indicates *p* < 0.05 as analyzed by a one-way ANOVA with a Bonferroni post hoc test). (**E**). Western blots following treatment with 50 nM rocaglamide A for 48 h in SUM-159PT cells. Band intensities were calculated by densitometry based on the ratio of each target gene (BIRC5 or ROCK1) normalized to the β-actin protein signal with 0 nM set to 100. (**F**). Western blots following treatment with 0–50 nM digoxin for 48 h in SUM-159PT cells. Band intensities were calculated based on the ratio of each target gene (BIRC5 or ROCK1) normalized to the β-tubulin protein signal with 0 nM set to 100. (**G**). Western blots following treatment with 50 nM of each indicated drug for 72 h in SUM-159PT cells. Band intensities were calculated based on the ratio of each target gene (eIF4A, eIF4G, or eIF4E) normalized to the β-tubulin protein signal with 0 nM set to 100. Uncropped Blots are shown in Figure S2. (**H**). Quantitation of mRNA levels by qPCR from SUM-159PT cells following the treatment of 50 nM digoxin. Data were analyzed using the ΔΔCt method with β-tubulin as the control. Relative expression was calculated with DMSO-treated cells set to 100% (* indicates *p* < 0.05 as analyzed by a one-way ANOVA with a Bonferroni post hoc test).

We further refined the results of our Prestwick screen by investigating the concentration-dependent reduction of (CGG)_4-_Luc2-tdTomato expression in the three TNBC cell lines (Figure 3A). All three cell lines showed favorable (CGG)_4_ RFU IC_50_ values ranging from 40–90 nM for digoxin (Figure 3B). Previous studies with digoxin have suggested that this compound can significantly inhibit global protein synthesis as inhibition of the Na^+^, K^+^-ATPase depletes cellular potassium which is required for translational elongation [34]. Based on the results of this study, we re-examined our findings from the (CGG)_4_ reporter assay.

To address the toxicity concerns associated with digoxin, we repeated the experiment with 231-S cells, but also normalized the fluorescent readings to total protein values of each well. With total protein normalization, both rocaglamide A and digoxin showed similar (CGG)_4_ RFU IC_50_ values (Figure 3C). Next, we looked at the relative luciferase activity of CMV-luciferase in HEK-293 cells as a generic indicator of total protein synthesis. Concentrations up to 50 nM of digoxin did not have a significant impact on the luciferase activity levels. However, concentrations exceeding 50 nM of digoxin did significantly reduce firefly luciferase activity levels (Figure 3D). These results would support previous reports that digoxin does indeed affect global protein synthesis at high concentrations. However, based on our (CGG)_4_ IC_50_ values, we believe that a therapeutic window does exist to target eIF4A1 without significant reductions in global protein synthesis. To support this claim, we first confirmed the dependency of two eIF4A targets (BIRC5 and ROCK1) in SUM-159PT cells by treating the cells with rocaglamide A. Levels of BIRC5 and ROCK1 decreased after 48 h of RocA treatment (Figure 3E). We then treated SUM-159PT cells with 30–50 nM digoxin and observed ROCK1 and BIRC5 decrease in the same time duration of drug treatment, but not β-tubulin (Figure 3F). Finally, to investigate a potential mechanism by which cardiac glycosides inhibit eIF4A1-mediated translation, we examined the expression levels of all three eIF4F components. Rocaglamide A is known to inhibit eIF4A1 by inducing non-specific binding and clamping to polypurine RNA [18]. We therefore did not expect any reductions in eIF4A1 levels upon rocaglamide A treatment. Interestingly, both digoxin and bufalin significantly reduced protein levels of eIF4A1. Expression levels of eIF4G, but not eIF4E, were also reduced by digoxin or bufalin (Figure 3G). We next investigated if reductions in eIF4A protein levels were occurring at the transcriptional level. Digoxin treatment of SUM-159PT cells significantly reduced eIF4A1 mRNA levels along with c-MYC (Figure 3H). 

### 2.4. Cardiac Glycosides Modulate eIF4A1 Expression Levels through c-MYC

**Figure 4:**
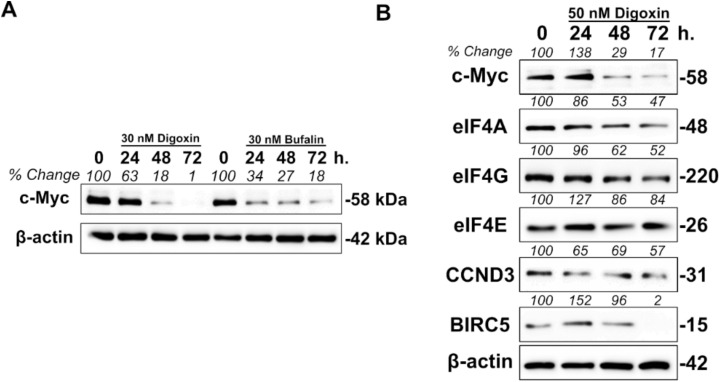
Cardiac glycosides modulate eIF4A expression levels through c-MYC (**A**). Western blot of BT20 cells following treatment with 30 nM of digoxin or bufalin for the indicated times. Band intensities were calculated by densitometry based on the ratio of c-MYC normalized to β-actin with 0 h set to 100 for each compound. (**B**). Western blot of SUM-159PT cells following treatment with 50 nM digoxin for the indicated times. Band intensities were calculated based on the ratio of each target normalized to β-actin with 0 h set to 100. Uncropped blots are shown in Figure S2

We next wanted to further elucidate the mechanism by which cardiac glycosides were decreasing the protein levels of eIF4A1. The Library of Integrated Network-Based Cellular Signatures (LINCS) is a National Institute of Health (NIH) sponsored program which catalogs proteomic and transcriptomic perturbation-response signatures across several cancer types. Integrated LINCS (iLINCS) is a biologist friendly extension of this program which allows for easy identification and analysis of these signatures. When examining biological processes affected by digoxin or bufalin in other systems, transcription was largely impacted [35,36]. Both digoxin and bufalin-treated MCF7 gene set enrichment analyses suggested that c-MYC was largely affected (Table 1 and Table 2). This data affirms previous reports that cardiac glycosides are known inhibitors of c-MYC [37,38]. To further validate this claim, we treated BT20 cells (another TNBC cell line) with only 30 nM of digoxin or bufalin and observed a time-dependent decrease in the levels of c-MYC (Figure 4A).

The c-MYC-eIF4F axis has been established to be an important mediator of tumorigenesis [39,40]. Moreover, the eIF4F complex and c-MYC are in a positive feedback loop with one another. Expression levels of c-MYC are regulated by the presence of secondary structure in its 5′-leader region. Components of the eIF4F complex are then in turn transcriptionally regulated by c-MYC [41]. Based on this literature and our own findings, we hypothesized that cardiac glycosides could inhibit eIF4A1-mediated translation through modulations in c-MYC levels. To test this hypothesis, we first examined at the number of MYC binding sites in the promoters of eIF4A1, eIF4E, and eIF4G. All three promoters contained putative MYC binding sites with eIF4A1 containing only one (Table 3). Next, we confirmed decreases in c-MYC and eIF4A levels in SUM-159PT cells upon treatment with digoxin. Cyclin D3 and BIRC5 levels also decreased in a time-dependent fashion suggesting that eIF4A1 was indeed affected. Moreover, the levels of eIF4G, but not eIF4E also decreased (Figure 4B). Next, we tested a c-MYC/Max dimerization inhibitor that has shown anti-cancer activity in breast cancer xenografts [42]. Kj Pyr 9 did show a concentration-dependent (CGG)_4_ RFU reduction in 293-HA-CXCR4 cells, although the IC_50_ value (9.7 μM) was quite high (Figure S1). This could be attributed to “undruggable” nature of c-MYC, a problem that the field has faced for decades [43]. Lastly, to support the specificity of our proposed hypothesis, we employed the ’forced overexpression’ of eIF4A1 or c-MYC in 293-HA-CXCR4 cells with bufalin treatment. Overexpression of either eIF4A or c-MYC significantly reduced the reduction of (CGG)_4_ Luc2-tdTomato by bufalin at high concentrations (Figure S1).

### 2.5. The Combination of Cardiac Glycosides and Rocaglates are Synergistic in Inhibiting TNBC Cells in Vitro

**Figure 5:**
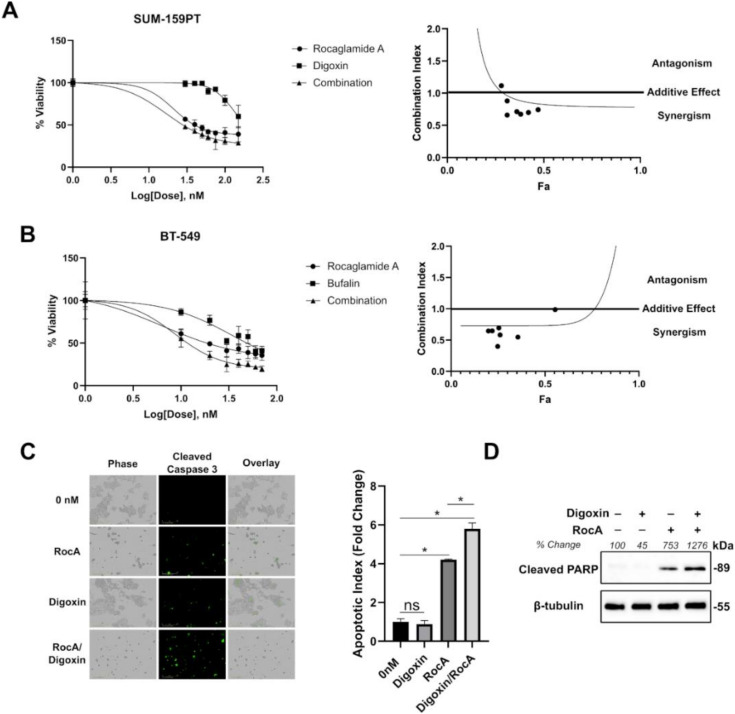
Rocaglates in combination with cardiac glycosides are synergistic in inhibiting TNBC cells in vitro. (**A**). Cell viability readings using Cell Titer GLO in SUM-159PT cells following the treatment of rocaglamide A, digoxin, or the combination of both (0–150 nM). (**B**). Cell viability readings using Cell Titer Blue in BT-549 cells following the treatment of rocaglamide A, bufalin, or the combination of both (0–70 nM). (**C**). Cleaved caspase 3 staining in SUM-159PT cells following treatment with rocaglamide A, digoxin, or the combination of both (50 nM) after 24 h. Apoptotic index was calculated based on the total green object integrated intensity normalized to the % confluence of each image (* indicates *p* < 0.05 as analyzed by a one-way ANOVA with Bonferroni post hoc test). (**D**). Western blots following treatment with 50 nM of digoxin, rocaglamide A, or the combination of both for 48 h in SUM-159PT cells. Band intensities were calculated by densitometry based on the ratio of cleaved poly ADP ribose polymerase (PARP) normalized to the β-tubulin protein signal with 0 nM set to 100. Uncropped blots are shown in Figure S2.

**Figure 6:**
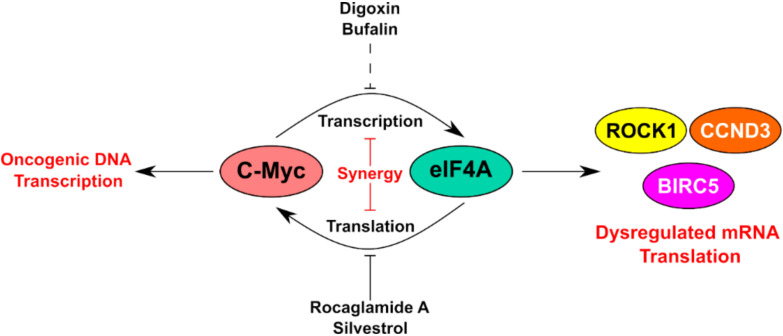
Proposed combinatorial targeting of eIF4A in TNBC cells. Inhibition of eIF4A, both directly and through modulation of upstream regulators (c-Myc), may prove synergistic as an anti-cancer therapy for triple-negative breast cancer patients.

Retrospective studies examining digoxin use in cancer patients has showed no survival advantages in recent years. However, digoxin usage does not increase mortality suggesting that it is safe to use in breast cancer patients at currently prescribed doses [44]. In addition, the toxicity of these compounds, especially against breast epithelial cells, has been raised by other groups [45]. Based on these findings, digoxin could be a good candidate for a combinatorial therapy in this population group especially at low-moderate doses. Due to the observation that digoxin could reduce total levels of eIF4A1, we hypothesized that cardiac glycosides could be synergistic with a known eIF4A1 inhibitor such as rocaglamide A. Cell viability experiments in SUM-159PT cells showed a synergistic effect with the combination of rocaglamide A and digoxin from 0–150 nM (Figure 5A). Moreover, to confirm that this phenomenon was not cell-type or digoxin-specific, we also tested the combination of rocaglamide A and bufalin in BT-549 cells from 0–70 nM (Figure 5B). The eIF4F complex regulates the mRNA translation of several anti-apoptotic proteins (like BCL-2 and myeloid cell leukemia 1 (MCL-1)) and rescues cells from caspase-mediated apoptosis [46]. We therefore visualized cleaved caspase 3/7 in live SUM-159PT cells. The combination of rocaglamide A and digoxin resulted in more caspase 3/7 cleavage over that of rocaglamide A alone (Figure 5C). To confirm our caspase 3/7 staining data, we also probed for an additional apoptotic marker. The combination of digoxin and rocaglamide A resulted in the largest increase of cleaved PARP following drug treatment (Figure 5D). In all, our results suggest that targeting of the c-MYC-eIF4A axis could be a beneficial and synergistic combination for TNBC patients (Figure 6).

## 3. Discussion

In this study, we identified several cardiac glycosides such as lanatoside C, digoxin, digoxigenin, and digitoxigenin to reduce the activity of eIF4A1. We continued the study with digoxin as its anti-cancer properties are under current clinical investigation (NCT03928210). We also employed bufalin to indicate that our findings were not digoxin specific. In our study, we found that digoxin and other cardiac glycosides are capable of reducing the total protein levels of eIF4A1. This was reflected in the loss of viability of several TNBC cell lines. Based on our data and dosing range, we did not observe a significant induction of apoptosis with digoxin treatment alone. However, when digoxin was combined with RocA, the level of induction of cleaved caspase-3 was further enhanced. This was confirmed with cleaved PARP, a second apoptotic marker.

Interestingly, digoxin has also been suggested to affect other biological processes such as anoikis and cellular signaling. In a screen of over 2000 off-patent drugs and natural products, digoxin was identified to induce cell death in anoikis-resistant suspension cultures of prostate adenocarcinoma cells [47]. Digoxin also appears to affect prominent oncogenic signaling nodes such as the cytosolic tyrosine kinase c-Src [48,49]. Recently, there has been renewed interest in digoxin for its ability to target senescent tumor cells. Cardiac glycosides were also identified to induce senolytic activity in cancer cells. The combination of senogenic (doxorubicin) and senolytic (digoxin) compounds showed remarkable synergy in inhibiting a patient-derived xenograft model of breast cancer [50]. Digoxin monotherapy was demonstrated to reduce the lymphatic dissemination in a xenograft model of breast cancer [51]. 

Here, we demonstrate that there was an observed decrease in both the mRNA and protein levels of eIF4A1 following digoxin treatment. This could be due to the observed decrease in the levels of the transcription factor c-MYC. MYC is known to transcriptionally regulate the protein level of eIF4A1 [41]. In support of our observations, an innovative high-throughput screen of various compounds identified cardiac glycosides as potent inhibitors of c-MYC expression. This was determined with a CRISPR-Cas9 engineered multiple myeloma cell line which had one allele of c-MYC tagged with GFP [38]. Importantly, cardiac glycosides were shown to inhibit the expression of c-MYC at the transcriptional level and we also confirmed this finding. Moreover, the ability of bufalin (a derivative of digoxin) to reduce c-MYC levels was demonstrated in xenograft models of pancreatic cancer [52]. 

There is a long-standing interest in the role of c-MYC and regulation of translational initiation [53]. For breast epithelium, c-MYC is required in the translational regulation of lactation and alveologenesis [54]. However, cancers are highly dependent on the activity of c-MYC to sustain mRNA translation [55]. More specifically, the eIF4F complex and c-MYC cooperate to promote tumorigenesis. Earlier studies found that c-MYC and eIF4E cooperate and facilitate immortalization of rat primary fibroblasts [56]. In cooperation with eIF4F, which helps to translate a variety of anti-apoptotic proteins, tumorigenesis is greatly accelerated [40]. Targeting of eIF4F in MYC-driven models of cancer greatly reduces tumor initiation [57]. Preventing the release of eIF4E by reducing phosphorylation levels of 4E-BP1 with mTOR inhibitors has also been another approach [58]. The c-MYC-eIF4E axis has also been extended to cancer immunotherapy. Reductions in p-eIF4E level with a MNK1/2 inhibitor reduced the expression of PD-L1 in a MYC/KRAS model of cancer [59]. Recently, c-MYC has also been shown to control 4E-BP1 levels [60]. This could be one translational control mechanism to reduce MYC-induced cellular stress.

Based on our data and the findings in the literature, we believe that a therapeutic window does exist to target eIF4A1 with cardiac glycosides. This comes from a growing evidence that in addition to inhibiting the Na^+^/K^+^-ATPase, cardiac glycosides also significantly impact the transcriptional regulation of proteins. Bufalin was shown to directly bind and promote the proteasome-mediated degradation of the steroid receptor coactivator (SRC) family of transcription factors [32]. Interestingly, SRC-1 was demonstrated to interact with Ets2 to increase c-MYC mRNA levels [61]. We showed that digoxin or bufalin treatment resulted in significant reductions of expression levels of both eIF4A1 and c-MYC. This is most likely occurring at the transcriptional level. Consistently, of the three eIF4F components, eIF4A1 was the most sensitive to a reduction by these compounds. This could be due to the fact that eIF4A only contains one c-MYC binding site in its promoter whereas eIF4E and eIF4G contain two sites. 

Despite numerous studies suggesting that digoxin and other cardiac glycosides are beneficial for the treatment of cancer, epidemiological studies have yielded inconsistent results. This could be due to the narrow therapeutic window of these compounds. In other words, efficacious concentrations cannot be obtained in the tumor without considerable toxicity in the patient. Due to the structural similarity to estrogen, several studies have suggested that digoxin may actually increase the risk for estrogen receptor positive breast cancer [62,63]. Based on these findings, we hypothesized that digoxin may be a good candidate at lower doses in combinatorial therapies for TNBC patients. The combination of rocaglamide A and digoxin or bufalin was shown to be synergistic in inhibiting the viability of TNBC cells. Inhibiting expression levels of eIF4A1 would thereby require decreased doses of rocaglamide A. Disruption of the c-MYC-eIF4F positive feedback loop could explain the synergistic effect. These findings could have future implications for Zotatifin (eFT226), a rocaglate currently in phase 1 clinical trials (NCT04092673). 

## 4. Materials and Methods

### 4.1. Cell Culture

MDA-MB-231 breast cancer cells were previously sorted for high cell surface expression of CXCR4 (denoted as 231-S) [64]. Human embryonic kidney 293 cells stably expressing human CXCR4 (denoted 293-HA-CXCR4) are also previously described [64]. MDA-MD-Bone-un cells are a bone metastatic variant of MDA-MB-231 cells [65]. Finally, 293-Parental, SUM-159PT, BT-20, and BT-549 cells were used in this study. Cells were maintained in high glucose Dulbecco’s Modified Eagle Medium (DMEM) (GE Healthcare Life Sciences, Pittsburgh, PA, USA, Cat. No. SH30243.01), 10% heat-inactivated fetal bovine serum (Denville Scientific, Swedesboro, NJ, USA, Cat. No. FB5001-H), and 1% Penicillin/Streptomycin solution (Corning, Corning, NY, USA, Cat. No. 30-002-CI).

### 4.2. Plasmids

Construction of the plasmids used in the (CGG)_4_ luciferase assay were described previously [66]. To create an eIF4A reporter gene, the (CGG)_4_ motif was inserted upstream of the Luciferase-dtTomato fusion protein. pCDH-2A-Luc2-dtTomato was a gift from Dr. Shi-He Liu (University of Toledo, Toledo, OH, USA). Previously, Luc2-dtTomato was inserted into the backbone of pCDH-EF1α-MCS-T2A-Puro (Systems Biosciences, Palo Alto, CA, USA, Cat. No. CD527A-1). The (CGG)_4_ motif was amplified from the CMV GQ 5′UTR luciferase plasmid by using the following primers: 5′-CTAGCTTCTAGACTAGGTTGAAAGTAC-3′ and 5′-AGCTAGGAATTCTTTACTATTCTAATCCG-3′. The (CGG)_4_ motif was then incorporated into the pCDH plasmid using added XbaI (5′end) and EcoRI (3′end) restriction sites. The open reading frame of eIF4A1 was a gift from Dr. Nahum Sonenberg (Mcgill University, Montreal, QC, Canada). To create an overexpression vector, eIF4A1 was amplified using the following primers: 5′-CTAGCTGGATCCACC ATGTCTGCGAGTCAGGATTC-3′ and 5′-CTAGCTGCGGCCGC TCAAATGAGGTCAGCAAC-3′. eIF4A1 was inserted into pcDNA3.0 (Invitrogen, Carlsbad, CA, USA, Cat. No. V79020) using added BamHI (5′end) and NotI (3′end) sites. pcDNA3.0-c-MYC was a gift from Dr. Nagalakshmi Nadiminty (University of Toledo, Toledo, OH, USA). 

### 4.3. Compounds and Drug Treatments

All compounds were purchased from MedChemExpress (Monmouth Junction, NJ, USA) and dissolved in DMSO. This includes rocaglamide A (Cat. HY-19356), silvestrol (Cat. HY-13251), mitoxantrone (HY-13502), paclitaxel (HY-B0015), digoxin (HY-B1049), and bufalin (HY-N0877). Drug containing media was only added at the beginning of the experiment. “0 nM” in all experiments signifies the DMSO control. If multiple concentrations were used, the amount of DMSO was matched to the highest drug condition.

### 4.4. Luciferase Assays

A total of 50 ng of each (CMV, CMV random (CGG)_4_, or CMV (CGG)_4_) pGL4.10 luciferase (Promega, Madison, WI, USA, Cat. No. E6651) was transfected along with 50 ng of pGL4.74 hRluc (Promega, Madison, WI, USA, Cat. No. E6921) using FuGENE 6 (Promega, Madison, WI, USA, Cat. No. E2691) in 293 cells using the manufacturer’s instructions. Firefly and renilla luciferase activity were taken 48 h later by using the Dual-Luciferase^®^ Reporter Assay System (Promega, Madison, WI, USA, Cat. No. E1910) and a SpectraMax iD5 plate reader.

### 4.5. Generation of (CGG)_4_ Reporter Cell Lines

The (CGG)_4_-Luc2-tdTomato fusion protein was stably inserted into the genome of MDA-MB-231-S, MDA-MB-Bone-un, SUM-159PT, and 293-HA-CXCR4 by lentiviral transduction. Virus was first packaged into 293-FT cells. T75 flasks were transfected with 4 μg of psPAX2, 2 μg pMD2.G, and 7 μg of pCDH-2A-(CGG)_4_-Luc2-dtTomato using Fugene 6 (Promega, Madison, WI, USA, Cat. E2691) according to the manufacturer’s instructions. Lentivirus was collected at the 48- and 72-h time points. Viral samples were concentrated using Amicon^®^ Ultra-15 Centrifugal 10 kDa Filter Units (Millipore Sigma, St. Louis, MO, USA, Cat. UFC901024). Lentivirus was added to cells overnight along with polybrene (8 μg/mL) (Millipore Sigma, St. Louis, MO, USA, Cat. H9268). The next day media was removed, and cells were continually selected in 2 μg/mL of puromycin (Millipore Sigma, St. Louis, MO, USA, Cat. P8833).

### 4.6. (CGG)_4_-Luc2-tdTomato and Total Protein Readings

Reporter cells were seeded into black/clear bottom 96-well plates and allowed to attach overnight (Corning, Oneonta, NY, USA, Cat. 3603). Full-serum and drug-containing media was then added the next day. Cells were incubated for 48 hours at 37 °C/5% CO_2_. Each well was then washed one time with 1× phosphate-buffered saline, pH 7.5 (PBS) and additional PBS was added for each reading. Fluorescent measurements were taken on a SpectraMax iD5 plate reader (Molecular Devices, San Jose, CA, USA). The 541_excitation_/581_emission_ well-scan readings were taken with 9 data points in each well (pattern: fill/density: 3/point spacing: 1.90). For plates with total protein readings, PBS was replaced with 1 × PBS/0.1% IGEPAL CA-630 (Millipore Sigma, St. Louis, MO, USA, Cat. 56741) and nutated for 20 min. Protein Bradford reagent (Bio-Rad, Hercules, CA, USA, Cat. 5000006) was added to each well. A595 readings were taken on the SpectraMax iD5 plate reader with standard endpoint settings.

### 4.7. Prestwick Chemical Library Screen

The Prestwick Chemical Library was a gift from Dr. Kevin Pan (University of Toledo, Toledo, OH, USA). Compounds were tested at a final concentration of 5 μM for 48 h before (CGG)_4_ RFU readings were obtained. The Z-score was calculated for each compound. More specifically, the following equation was used: [(CGG)_4_ RFU Compound X-(CGG_4_) RFU average of each 96 well plate]/(CGG)_4_ RFU standard deviation of the 96-well plate.

### 4.8. Western Blotting

Cell lysates were prepared by lysing the cells in the following buffer: 50 mM Tris pH 8.0, 100 mM NaCl, 0.1% IGEPAL CA-630, 0.1% Deoxycholate, 5 mM EDTA, protease inhibitor cocktail (Sigma-Aldrich, St. Louis, MO, USA, Cat. No. P8340-5ML), phosphatase inhibitor cocktail 2 (Sigma-Aldrich, St. Louis, MO, USA, Cat. No. P5726), and phosphatase inhibitor cocktail 3 (Sigma-Aldrich, St. Louis, MO, USA, Cat. No. P0044). Lysates were nutated for 10 min at 4 °C and centrifuged for 10 min at 10,000 RPM. Supernatants were quantified using protein bradford reagent and loaded onto an SDS-PAGE gel. Following transfer onto nitrocellulose, membranes were incubated with the following primary antibodies: eIF4A1 (Cell Signaling Technology, Danvers, MA, USA, Cat. 2490), eIF4E (Cell Signaling Technology, Danvers, MA, USA, Cat. 2067), eIF4G (Cell Signaling Technology, Danvers, MA, USA, Cat. 2469), BIRC5 (Cell Signaling Technology, Danvers, MA, USA, Cat. 2808), CCND3 (Cell Signaling Technology, Danvers, MA, USA, Cat. 2936), c-MYC (Proteintech, Rosemont, IL, USA, Cat. 10828-1-AP), cleaved PARP (Asp214) (Cell Signaling Technology, Danvers, MA, USA, Cat. 5625), β-actin (Cell Signaling Technology, Danvers, MA, USA, Cat. 8457), and β-tubulin D66 (Millipore Sigma, St. Louis, MO, USA, Cat. No. T0198). Following primary antibody incubation, goat anti-Mouse IgG (H + L) Superclonal™ Secondary Ab conjugated to HRP (Thermo Scientific, Rockford, IL, USA, Cat No. A28177) or Goat anti-Rabbit IgG (H + L) Superclonal™ Secondary Ab conjugated to HRP (Thermo Scientific, Rockford, IL, USA, Cat No. A27036) was used. Blots were developed with Amersham™ ECL™ Prime Western Blotting Detection Reagent (GE Healthcare Life Sciences, Pittsburgh, PA, USA, Cat. No. RPN2232) and a G-Box Chemi system (Syngene USA, Frederick, MD, USA). Densitometry was performed using ImageJ software.

### 4.9. qPCR

Total RNA was extracted from SUM-159PT cells using a RNeasy^®^ Mini Kit according to the manufacturer’s instructions (Qiagen, Germantown, MD, USA, Cat. No. 74104). Following RNA isolation, cDNA was synthesized using a ProtoScript^®^ II First Strand cDNA Synthesis Kit (NEB, Ipswich, MA, USA, Cat. No. E6560S). Then, 1 μg of input RNA and oligo-dT primers were used according to the manufacturer’s instructions. Finally, real-time PCR was performed using Luna^®^ Universal qPCR Master Mix (NEB, Ipswich, MA, USA, Cat. No. M3003L), 1 μL of cDNA, and the following forward and reverse primers: 1.) eIF4A1: 5′-AAGGCGTCATCGAGAGTAACT-3′ and 5′-ATGTGGCCGTTTTCCCAGTC-3′ 2.) c-MYC: 5′-CTTCTCTCCGTCCTCGGATTCT-3′ and 5′-GAAGGTGATCCAGACTCTGACCTT-3′ 3.) β-tubulin: 5′-TTGGCCAGATCTTTAGACCAGACAAC-3′ and 5′-CCGTACCACATCCAGGACAGAATC-3′. Real time data were analyzed using the ΔΔCt method with β-tubulin as the control. The values from DMSO treated SUM-159PT cells were then set to 100%.

### 4.10. Promoter Binding Analysis

The promoter sequence (−499 to +100) for eIF4A (ENSG00000161960), eIF4E (ENSG00000151247), and eIF4G (ENSG00000114867) was obtained from the Eukaryotic Promoter Database (https://epd.epfl.ch//index.php). Putative transcription factor binding sites were predicted by PROMO (http://alggen.lsi.upc.es/cgi-bin/promo_v3/promo/promoinit.cgi?dirDB=TF_8.3). Transcription factors were predicted within a dissimilarity margin less or equal than 15%. Only human factors and sites were considered for analysis.

### 4.11. iLINCS GSEA

The Integrative Library of Integrated Network-Based Cellular Signatures (iLINCS) was used to obtain transcriptomic data of digoxin and bufalin treated MCF7 cells (http://www.ilincs.org/ilincs/). More specifically, LINCSCP_33452 (digoxin) and LINCSCP_33779 (bufalin) signatures were chosen for further analysis. MCF7 cells were treated with 10 μM of each compound for 6 hours. Gene set enrichment analyses were performed by Enrichr. The “TF Perturbations Followed by Expression” gene-set library was selected for each compound.

### 4.12. Rescue Experiments

A total of 200 ng/well of pcDNA3-eIF4A1 or pcDNA3-c-MYC was transfected into 293-HA-CXCR4 (CGG)_4_ Luc2-tdTomato cells using Fugene 6 according to the manufacturer’s instructions. Following transfection, each indicated amount of bufalin added to each well. Cells were incubated for 48 h before taking (CGG)_4_ RFU and total protein (A595) readings.

### 4.13. Cell Viability

Cellular Viability was determined by using either Cell Titer-Glo (Promega, Madison, WI, USA, Cat. No. G7570) or Cell Titer-Blue (Promega, Madison, WI, USA, Cat. No. G8080) according to the manufacturer’s instructions. Then, 3000 cells were plated in each well and were allowed to attach overnight. The next day, DMEM was replaced with drug-containing media and readings were taken 48 h later.

### 4.14. Synergy Analysis

The combination index of each drug combination was calculated by using the Chou-Talalay method of drug synergy and CompuSyn software (www.combosyn.com) [67]. Data were obtained from CompuSyn and graphed in Prism.

### 4.15. Live-cell Cleaved Caspase 3 Staining

Cleaved caspase 3/7 was stained in live cells by using the IncuCyte caspase 3/7 green reagent for Apoptosis (Essen BioScience, Ann Arbor, MI, USA, Cat. 4440) according to the manufacturer’s instructions. Images were captured in an IncuCyte system (Essen Biosciences, Ann Arbor, MI, USA). Data were analyzed by calculating the total green object integrated intensity and % confluence of each image using the IncuCyte software.

### 4.16. Graph Preparation, Determination of IC_50_ Values, and Statistical Analysis

All graphs, IC_50_ values, and statistical analysis was performed by using Prism 8 (GraphPad Software, San Diego, CA, USA).

### 4.17. Patents 

Data in this manuscript have been included in a PCT patent application (60755-WO-PCT/D2019-32) in which Dayanidhi Raman, Amit K. Tiwari, and Cory M. Howard are listed as inventors. 

## 5. Conclusions

In summary, we have identified cardiac glycosides as potential inhibitors of eIF4A1-mediated translation. This occurs in a narrow therapeutic window via decreases in c-MYC and subsequent eIF4A1 levels. Retrospective studies have indicated that digoxin does not increase mortality in breast cancer patients. It therefore may be a good candidate in combination with other eIF4F inhibitors for the treatment of triple-negative breast cancer. 

## Figures and Tables

**Table 1:** Digoxin gene set enrichment analysis.

Term	Overlap	*p*-Value	Adjusted *p*-Value	Old *p*-Value	Old Adjusted *p*-Value	Odds Ratio	Combined Score
MYC oe MCF7 human gse101738 RNAseq down	11/159	4.36 × 10^−10^	8.54 × 10^−7^	0	0	13.83647799	298.2241
SREBF2 KD human gse50588 creedsid gene 2823 up	12/294	2.70 × 10^−8^	2.64 × 10^−5^	0	0	8.163265306	142.2611
STAT3 KD human gse42979 creedsid gene 2148 down	12/309	4.67 × 10^−8^	3.05 × 10^−5^	0	0	7.766990291	131.1075
HEY2 KO mouse gse6526 creedsid gene 1511 up	9/205	8.91 × 10^−7^	3.49 × 10^−4^	0	0	8.780487805	122.3245
HEY2 KO mouse gse6526 creedsid gene 1512 down	9/205	8.91 × 10^−7^	2.91 × 10^−4^	0	0	8.780487805	122.3245

**Table 2:** Bufalin gene set enrichment analysis.

Term	Overlap	*p*-Value	Adjusted *p*-Value	Old *p*-Value	Old Adjusted *p*-Value	Odds Ratio	Combined Score
MYC oe MCF7 human gse101738 RNAseq down	8/159	1.34 × 10^−6^	8.74 × 10^−4^	0	0	10.0628931	136.0840
ZNF143 siRNA MCF7 human gse76453 RNAseq down	10/233	2.63 × 10^−7^	5.15 × 10^−4^	0	0	8.58369099	130.0552
STAT3 deficiency mouse gse6846 creedsid gene 85 down	10/246	4.33 × 10^−7^	4.24 × 10^−4^	0	0	8.1300813	119.1174
FOXO1 KD mouse gse6623 creedsid gene 505 down	8/195	6.12 × 10^−6^	0.001711	0	0	8.20512821	98.4977
SREBF2 KD human gse50588 creedsid gene 2823 up	10/294	2.18 × 10^−6^	8.54 × 10^−4^	0	0	6.80272109	88.6777

**Table 3:** c-MYC putative promoter binding analysis.

Gene	Factor Name	Start Position	End Position	Dissimilarity	String	Random Expectation Equally	Random Expectation Query
eIF4A	c-MYC (T00140)						
		204	209	0.000000	CACGTG	0.14648	0.19526
eIF4E							
		266	271	0.000000	CACGTG	0.14648	0.16595
		419	424	0.000000	CACGTG	0.14648	0.16595
eIF4G							
		355	360	0.000000	CACGTG	0.14648	0.18917
		432	437	0.000000	CACGTG	0.14648	0.18917

## Supplementary Materials

The following are available online at https://www.mdpi.com/2072-6694/12/8/2169/s1, Figure S1: Cardiac glycosides modulate eIF4A expression levels through c-MYC; Figure S2: Uncropped blot figures.
