# Supplementary material for: Identification of Cardiac Glycosides as Novel Inhibitors of eIF4A1-Mediated Translation in Triple-Negative Breast Cancer Cells
Source: Cancers (Basel). 2020 Aug 4;12(8):2169. doi: 10.3390/cancers12082169 (PMC7465665; doi:10.3390/cancers12082169)
Supplement: Supplementary file 1 [file cancers-12-02169-s001.zip › cancers-867140-supplementary final/cancers-861740 supplementary layout.docx]

Supplementary Materials

Identification of Cardiac Glycosides as Novel Inhibitors of eIF4A1-Mediated Translation in Triple-Negative Breast Cancer Cells

Cory M. Howard, Matthew Estrada, David Terrero, Amit K. Tiwari and Dayanidhi Raman


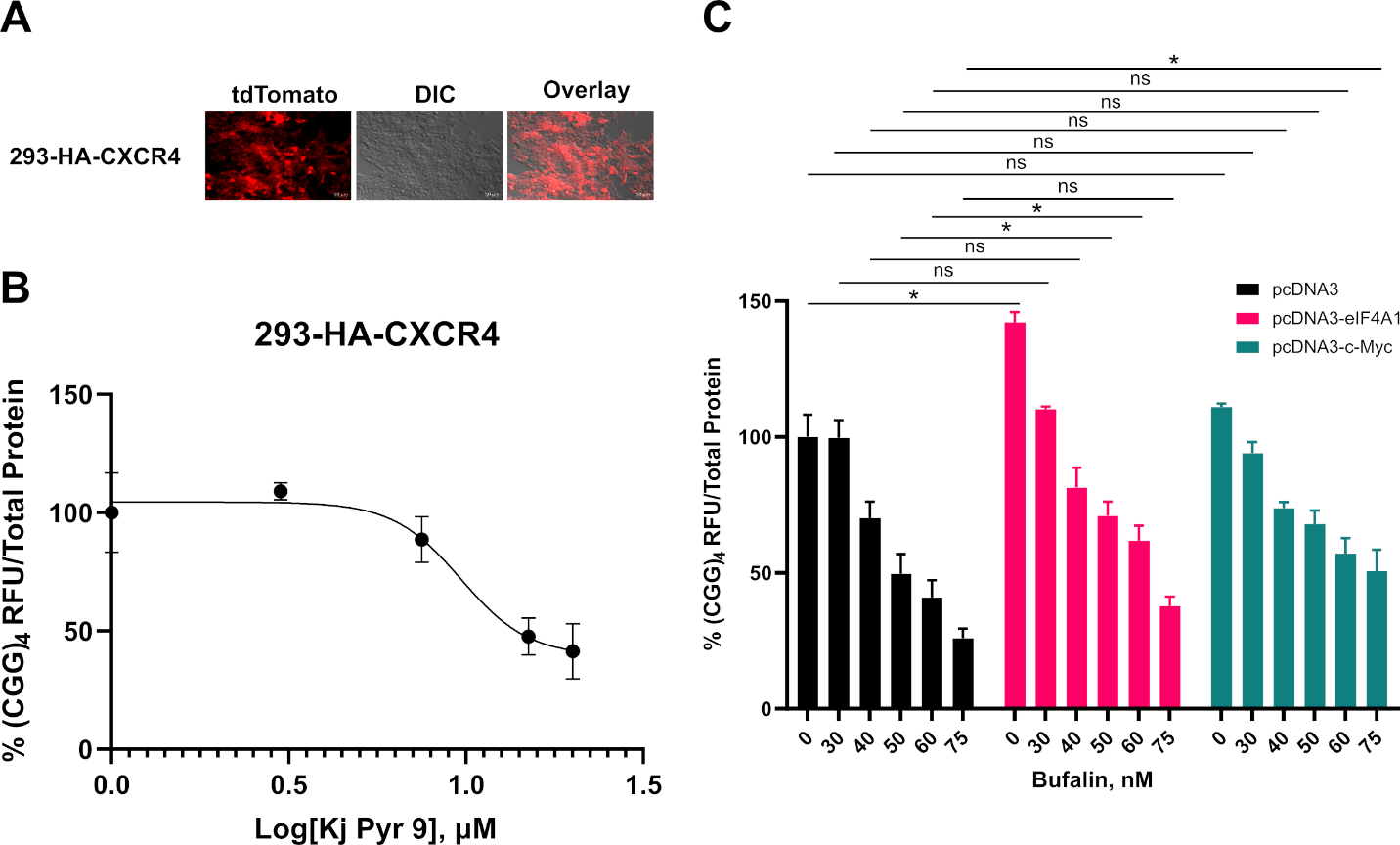


**Figure S1.** Cardiac glycosides modulate eIF4A expression levels through c-MYC. (**A**). Live-cell fluorescent images of 293-HA-CXCR4 cells after introduction of the (CGG)4 Luc2-tdTomato fusion protein (n = 1). (**B**). (CGG)_4_ Luc2-tdTomato/total protein readings (A595) in 293-HA-CXCR4 cells following treatment with 0–20 μM KJ Pyr 9. (**C**). (CGG)_4_ Luc2-tdTomato/total protein readings (A595) in 293-HA-CXCR4 cells following the transfection of pcDNA3-eIF4A1 or pcDNA3-c-MYC and 0–75 nM bufalin (* indicates *p* < 0.05 as analyzed by a one-way ANOVA with a bonferroni post hoc test).

| 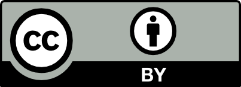 | © 2020 by the authors. Licensee MDPI, Basel, Switzerland. This article is an open access article distributed under the terms and conditions of the Creative Commons Attribution (CC BY) license (http://creativecommons.org/licenses/by/4.0/). |
| --- | --- |
