# Supplementary material for: Identification of Cardiac Glycosides as Novel Inhibitors of eIF4A1-Mediated Translation in Triple-Negative Breast Cancer Cells
Source: Cancers (Basel). 2020 Aug 4;12(8):2169. doi: 10.3390/cancers12082169 (PMC7465665; doi:10.3390/cancers12082169)
Supplement: Supplementary file 1 [file cancers-12-02169-s001.zip › cancers-867140-supplementary final/Figure S2. Uncropped blot figures.pdf]

Figure 3E

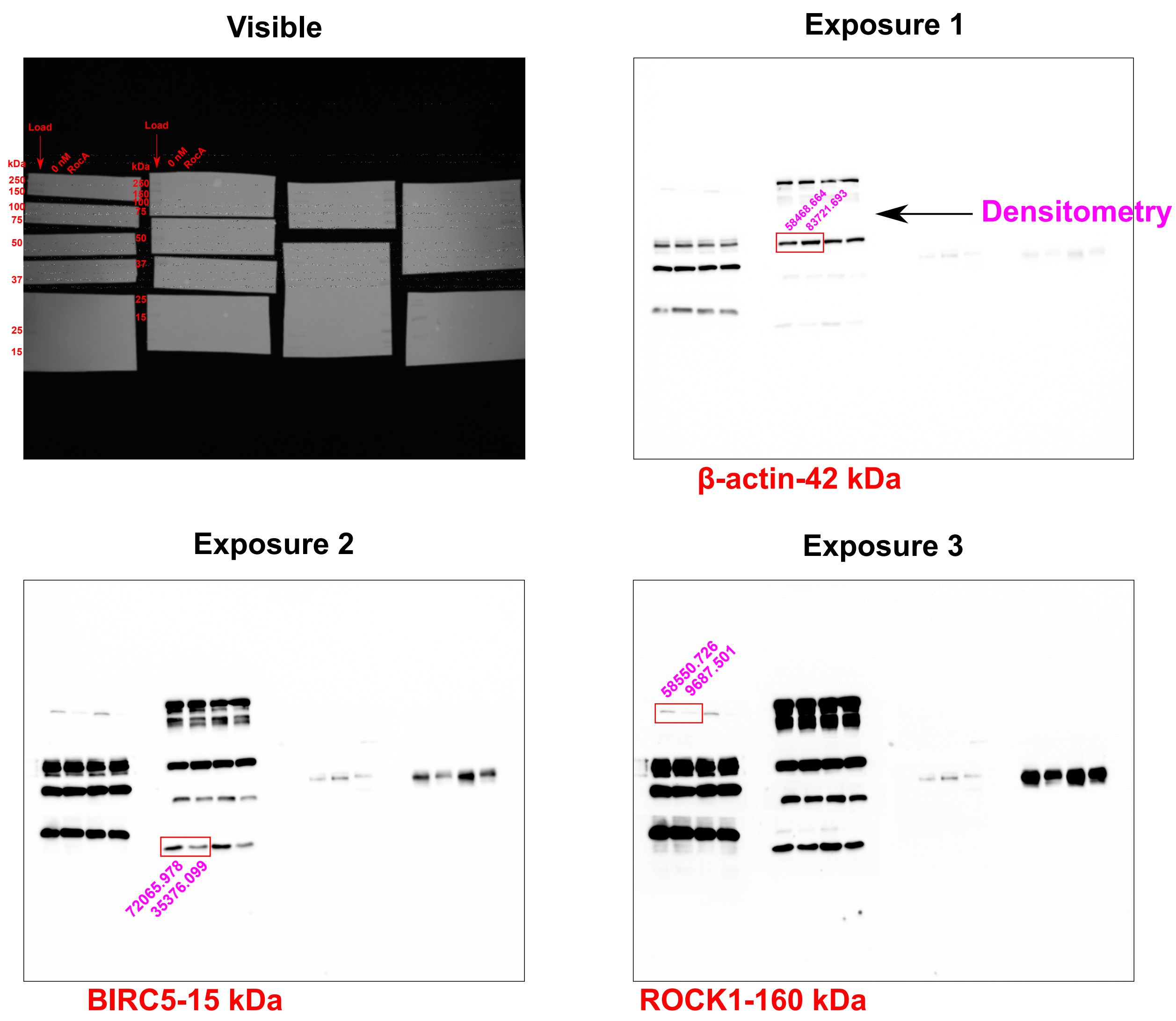

Visible
Load
Load
kDa
0 nM
RocA
250
150
100
75
50
37
25
15
Exposure 1
58468.664
83721.693
Densitometry
β-actin-42 kDa
Exposure 2
72065.978
35376.099
BIRC5-15 kDa
Exposure 3
58550.726
9687.501
ROCK1-160 kDa

Figure 3F

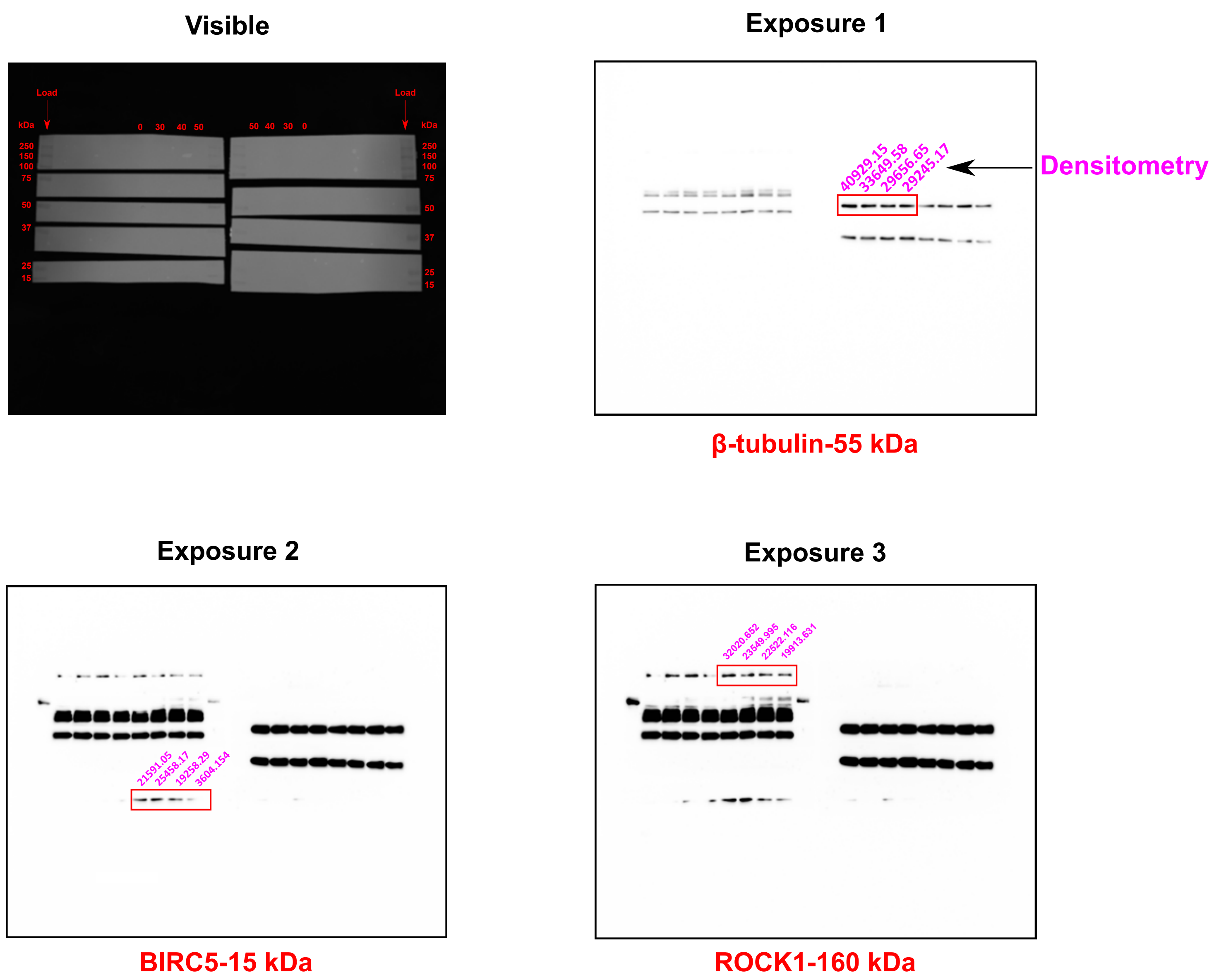

Visible
Load
kDa
250
150
100
75
50
37
25
15
0 30 40 50
50 40 30 0
Exposure 1
40929.15
33649.58
29656.65
29245.17
Densitometry
β-tubulin-55 kDa
Exposure 2
21591.05
25458.17
19258.29
3604.154
BIRC5-15 kDa
Exposure 3
32020.652
23549.995
22522.116
19913.631
ROCK1-160 kDa

Figure 3G

Visible

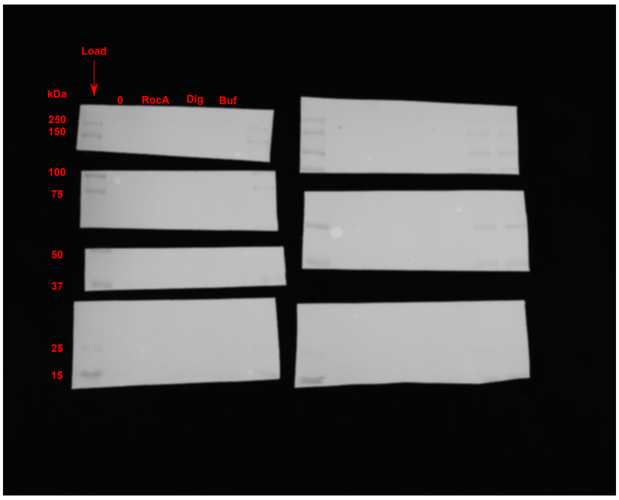

Exposure 1

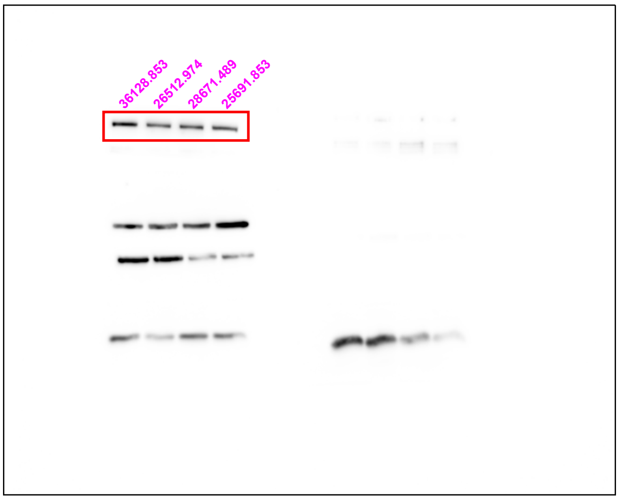

eIF4G-220 kDa

Exposure 2

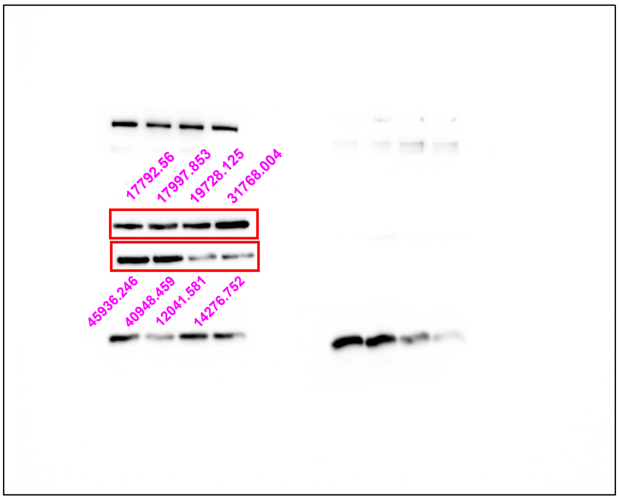

β-tubulin-55 kDa
eIF4A-48 kDa

Exposure 3

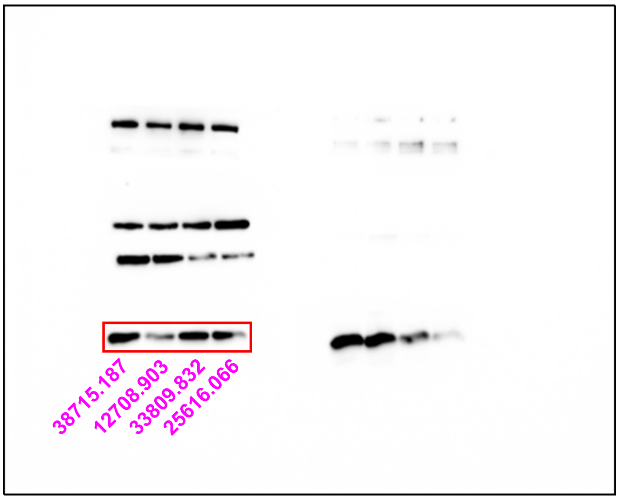

eIF4E-26 kDa

Figure 4A

Visible

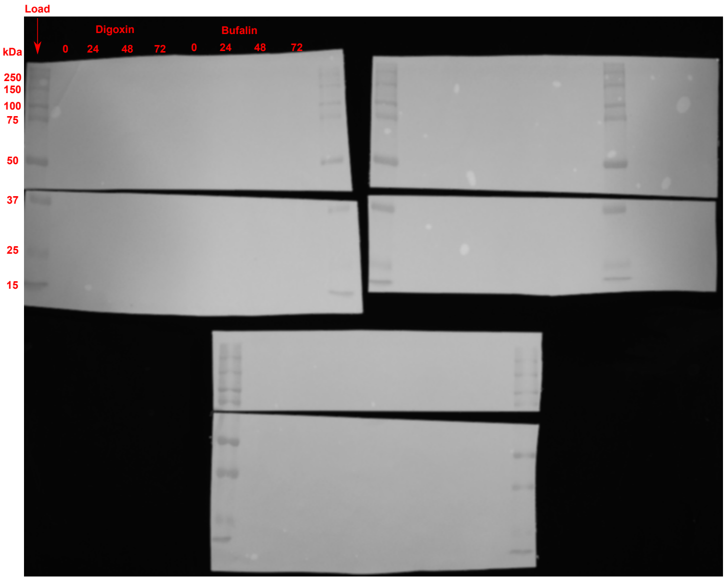

Exposure 1

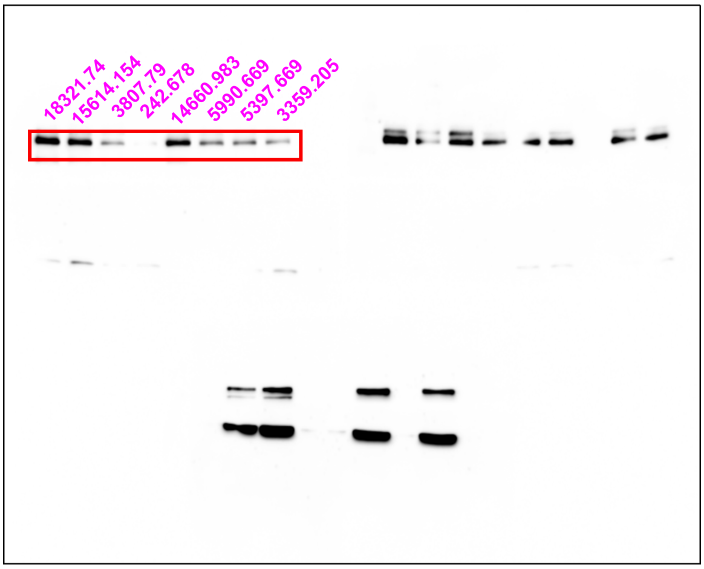

c-MYC-58 kDa

Visible

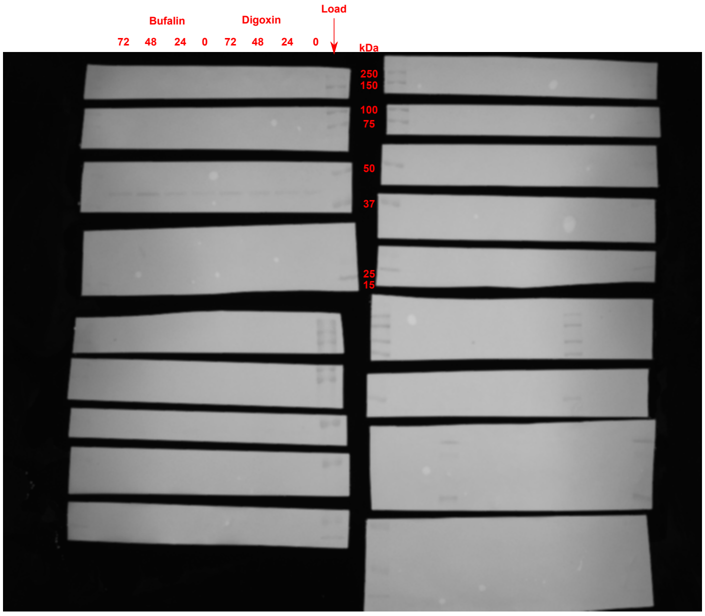

Exposure 1

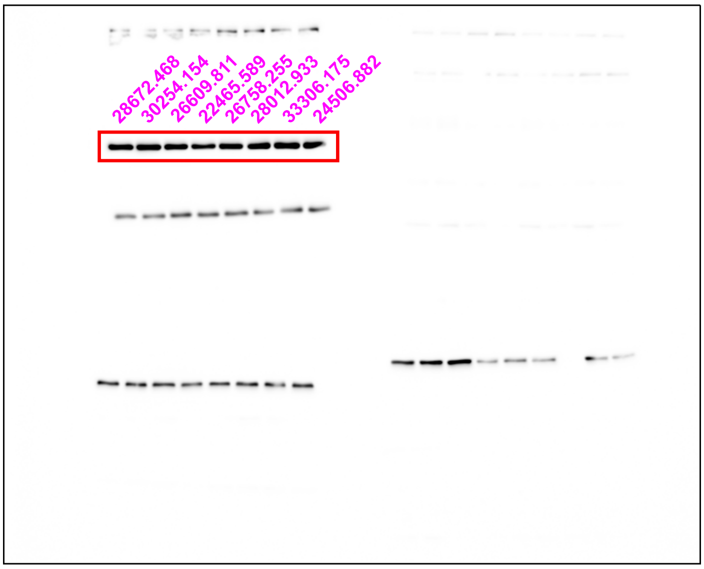

β-actin-42 kDa

Figure 4B

Visible

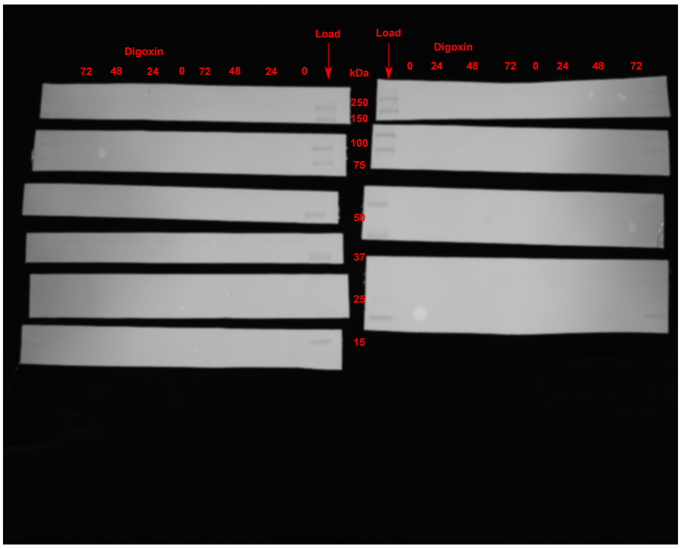

Exposure 1

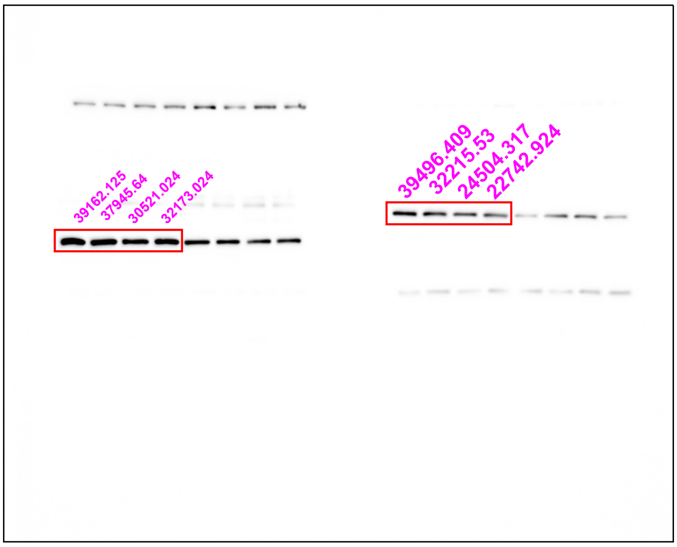

β-actin-42 kDa

eIF4A-48 kDa

Exposure 2

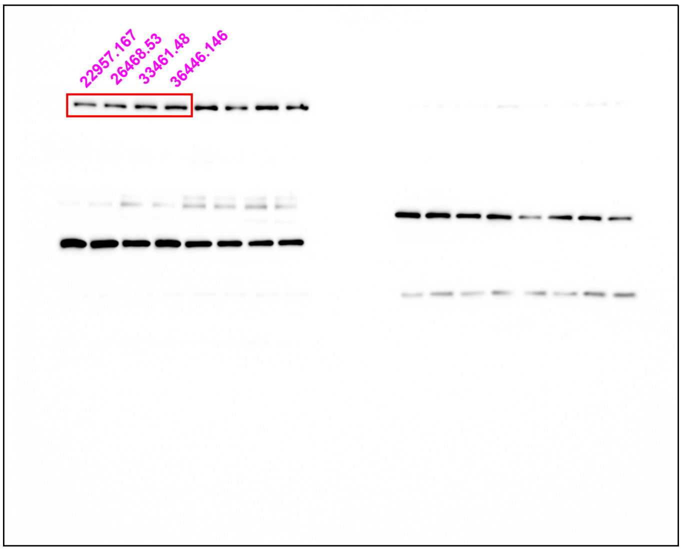

eIF4G-220 kDa

Exposure 3

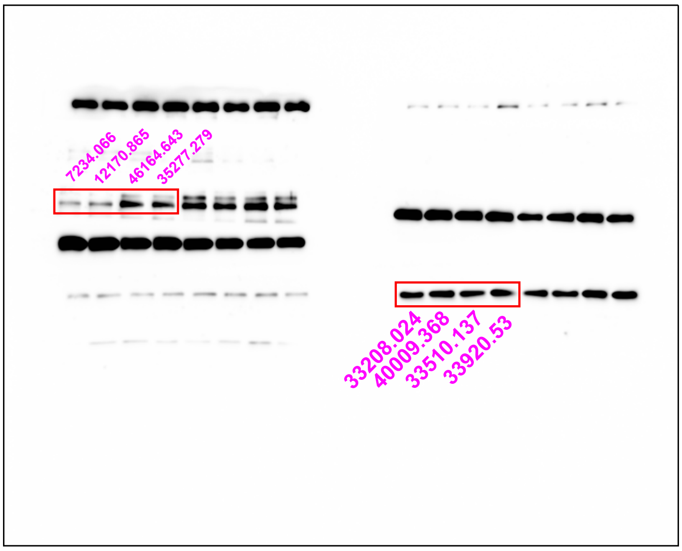

c-MYC-55 kDa

eIF4E-26 kDa

Exposure 4

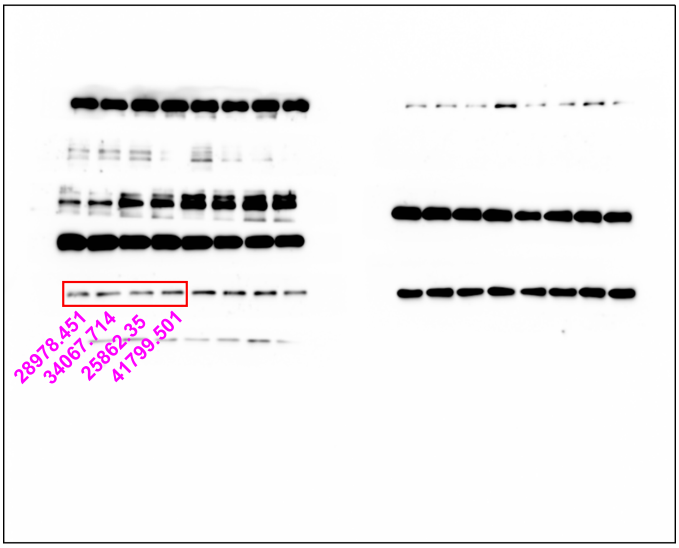

CCND3-31 kDa

Exposure 5

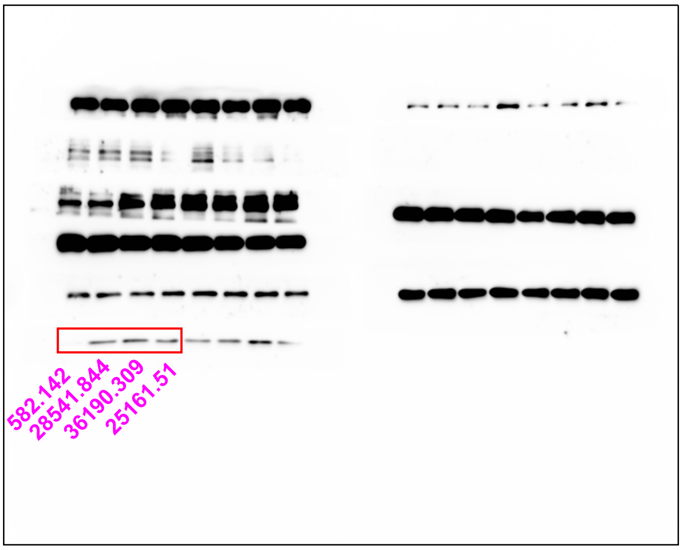

BIRC5-15 kDa

Figure 5D

Visible

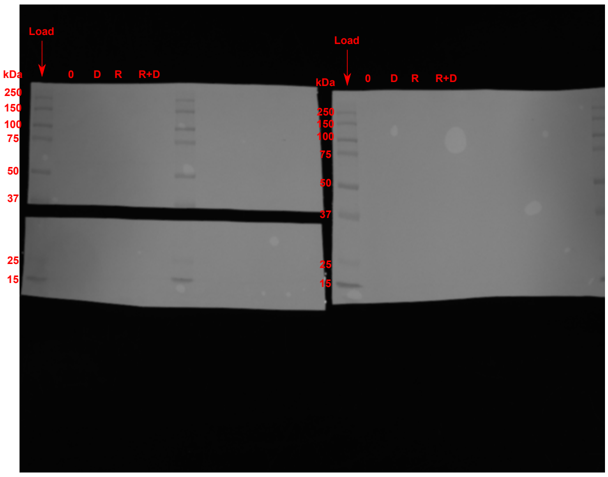

Exposure 1

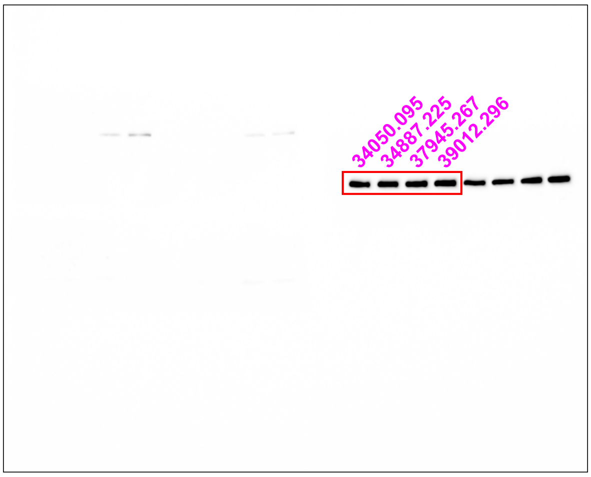

β-tubulin-55 kDa

Exposure 2

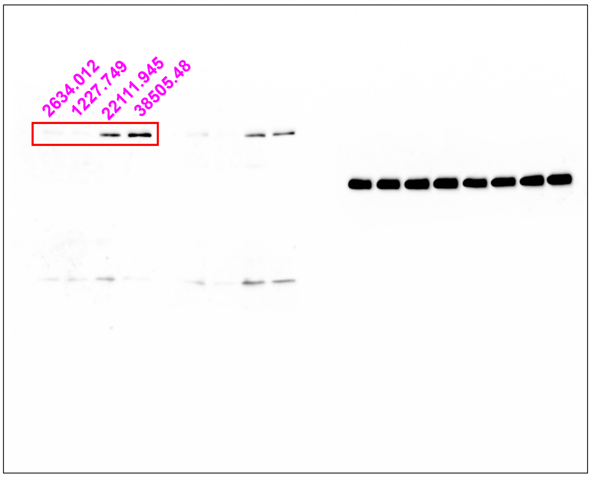

Cleaved PARP-89 kDa
